# Construction and Immunogenicity Evaluation of a Digestive Protein-Based Chimeric Antigen Composed of Cathepsin L1, Cathepsin B1, and Saposin-like Protein 2 from *Fasciola gigantica*

**DOI:** 10.3390/ijms27135724

**Published:** 2026-06-25

**Authors:** Werachon Cheukamud, Supanan Chansap, Komsil Rattanasroi, Narin Changklungmoa, Pornanan Kueakhai

**Affiliations:** 1 Research Unit of Vaccine and Diagnosis of Parasitic Diseases, Faculty of Allied Health Sciences, Burapha University, Long-Hard Bangsaen Road, Saen Sook Sub-District, Mueang District, Chonburi 20131, Thailand; 61810046@go.buu.ac.th (W.C.); supanan.cn@go.buu.ac.th (S.C.); komsil.r@vistec.ac.th (K.R.); narinchang@go.buu.ac.th (N.C.); 2 School of Biomolecular Science and Engineering, Vidyasirimedhi Institute of Science and Technology (VISTEC), Rayong 21210, Thailand

**Keywords:** fasciolosis, digestive protein, chimeric protein, immunization, *Fasciola gigantica*

## Abstract

Fasciolosis, caused by the liver fluke *Fasciola gigantica*, remains a major parasitic disease affecting livestock in tropical regions and results in substantial economic losses. Although anthelmintic drugs are widely used for disease control, increasing reports of drug resistance highlight the need for alternative strategies such as vaccination. In this study, a recombinant digestive protein-based chimeric antigen (rFgCHI-DP) composed of three *F. gigantica* antigens—cathepsin L1 (FgCL1), cathepsin B1 (FgCB1), and saposin-like protein 2 (FgSAP2)—was designed and expressed in *Escherichia coli*. The mature regions of these proteins were sequentially linked to form a single chimeric construct. The recombinant protein was successfully expressed and purified under denaturing conditions, producing a protein of approximately 62 kDa. To evaluate its immunogenicity, BALB/c mice were immunized with rFgCHI-DP formulated with Quil A adjuvant. Indirect ELISA revealed that immunization induced antigen-specific IgG responses. Antibody responses showed strong reactivity toward FgCL1 and FgCB1, whereas the response against FgSAP2 was comparatively lower. Western blot analysis further demonstrated that antibodies generated against rFgCHI-DP recognized native parasite antigens. Immunolocalization also revealed that the anti-rFgCHI-DP antibodies could detect targeted antigens in the cecal epithelium of the parasite. These findings indicate that the adult-stage chimeric protein rFgCHI-DP is immunogenic in mice and capable of inducing specific antibody responses against *F. gigantica*. The results support the potential of rFgCHI-DP as a candidate antigen for future fasciolosis vaccine development.

## 1. Introduction

Fasciolosis is a parasitic disease caused by the liver flukes *Fasciola hepatica* and *F. gigantica*, which infect a wide range of mammalian hosts including cattle, sheep, goats, and occasionally humans. The disease is widely distributed in tropical and subtropical regions and causes substantial economic losses in the livestock industry due to reduced productivity, liver condemnation, and treatment costs [1]. In many Asian and African countries, *F. gigantica* represents the predominant species responsible for infections in ruminants and remains a major challenge for parasite control [2]. Currently, fasciolosis is mainly controlled through the administration of anthelmintic drugs, particularly triclabendazole. However, increasing reports of drug-resistant *Fasciola* populations have raised concerns regarding the long-term effectiveness of chemotherapy-based control strategies [3,4]. In addition, drug treatment does not prevent reinfection, and repeated administration can increase the risk of resistance development. Consequently, the development of effective vaccines has been proposed as an alternative and sustainable approach for controlling fasciolosis [5].

Several parasite-derived molecules have been investigated as vaccine candidates against *Fasciola* infections. Among these, digestive proteins expressed in the parasite gut have attracted considerable interest because of their essential roles in host tissue penetration, blood feeding, nutrient acquisition, and parasite survival. Cathepsin L1 (CL1) and Cathepsin B1 (CB1), two major cysteine proteases secreted by juvenile and adult flukes, are key components of the parasite digestive system [6,7]. These enzymes are released into the gut lumen and excretory–secretory (ES) products, where they participate in the degradation of host proteins, including hemoglobin, serum albumin, immunoglobulins, and extracellular matrix components. In addition to facilitating nutrient uptake, CL1 and CB1 contribute to tissue invasion, immune modulation, and evasion of host defense mechanisms, making them attractive targets for vaccine development [8,9,10].

Another important digestive protein is Saposin-like protein 2 (SAP2), which is expressed from the juvenile to adult stages and is abundantly present in both the gastrodermal cells and ES products of *Fasciola* spp. SAP2 belongs to a family of lipid-binding and membrane-active proteins that possess membrane-lytic activity, enabling the disruption of host cell membranes, including erythrocytes. This activity facilitates access to digestive proteases to intracellular nutrients, thereby enhancing nutrient acquisition and supporting the proteolytic functions of cathepsins within the parasite gut. In addition, SAP2 is highly antigenic and continuously exposed to the host immune system through its secretion in ES products, making it a promising vaccine target [11,12,13,14,15,16].

Vaccination studies using recombinant cathepsins have demonstrated their ability to induce both humoral and cellular immune responses and to confer varying degrees of protection against *Fasciola* infections in experimental animals [7,17,18,19]. Among the cathepsins, CL1 is considered one of the most immunodominant and extensively characterized vaccine candidates due to its high expression levels in adult flukes and its central role in protein digestion. Likewise, CB1 functions synergistically with CL1 in the proteolytic cascade responsible for the breakdown of host-derived nutrients and has also shown promising immunoprotective properties. Collectively, CL1, CB1, and SAP2 play complementary roles in digestion, nutrient acquisition, and host–parasite interactions, supporting their selection as promising components for the development of multivalent vaccines against fasciolosis.

Collectively, the indispensable biological functions of CL1, CB1, and SAP2 in parasite feeding, digestion, and host–parasite interactions make these digestive proteins attractive candidates for vaccine development against fasciolosis. However, vaccines based on a single antigen often provide only partial protection because they target a limited range of parasite functions and immune epitopes.

Recent advances in vaccine development have increasingly focused on the design of chimeric proteins that combine multiple antigens within a single construct. By incorporating several immunologically relevant molecules, chimeric proteins can simultaneously present diverse antigenic determinants to the immune system, potentially broadening immune recognition and enhancing protective efficacy compared with single-antigen vaccines [20,21]. In our previous study, a juvenile-stage chimeric protein composed of FgCL1H, FgCB3, and FgSAP1 elicited strong humoral immune responses in mice and generated antibodies capable of recognizing native parasite antigens [22]. These findings support the feasibility of using multivalent chimeric antigens as vaccine candidates against *Fasciola* infections. Despite these encouraging results, relatively little attention has been given to chimeric vaccines based on digestive proteins that are expressed throughout the juvenile-to-adult developmental stages of *F. gigantica*. The adult fluke is responsible for chronic infection, continuous nutrient acquisition, and long-term hepatobiliary pathology in the definitive host. Consequently, targeting digestive proteins that are highly expressed and functionally essential during this stage may represent an effective strategy for impairing parasite survival, limiting nutrient utilization, and reducing disease progression.

In the present study, we designed and constructed a rFgCHI-DP comprising three major digestive proteins of *F. gigantica*: Cathepsin L1 (FgCL1), Cathepsin B1 (FgCB1), and Saposin-like protein 2 (FgSAP2). Following in silico characterization and heterologous expression in *Escherichia coli*, the immunogenicity of rFgCHI-DP was evaluated in BALB/c mice to assess its capacity to induce immune responses and its potential for future development as a multivalent vaccine candidate against fasciolosis.

## 2. Results

### 2.1. Antibody Epitope Predictions

**Figure 1:**
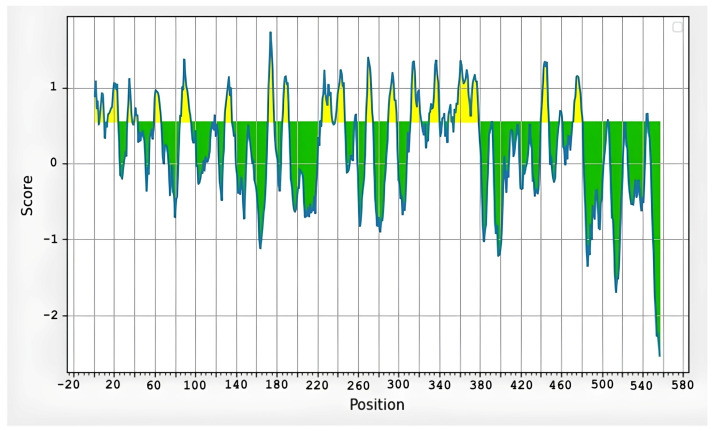
Predicted B-cell epitopes of rFgCHI-DP protein graph. Y-axes depicts residue scores and X-axes residue positions in the sequence. Prediction scores above 0.5 were considered B-cell epitopes (yellow), whereas scores below 0.5 were considered non-B-cell epitopes (green), (average score: 0.190 Minimum score: −0.002 Maximum score: 1.735).

Analysis of the rFgCHI-DP amino acid sequence using the BepiPred-2.0 server predicted 22 linear B-cell epitopes with scores greater than 0.5 (Figure 1). The predicted epitopes were distributed throughout the three antigenic components of the chimeric protein, including FgCL1, FgCB1, and FgSAP2. These findings indicate that all regions of the construct possess antigenic determinants capable of being recognized by B cells and may therefore contribute to the induction of humoral immune responses (Table 1).

### 2.2. Construction of the rFgCHI-DP Gene of FgCL1, FgCB1, and FgSAP2

**Figure 2:**
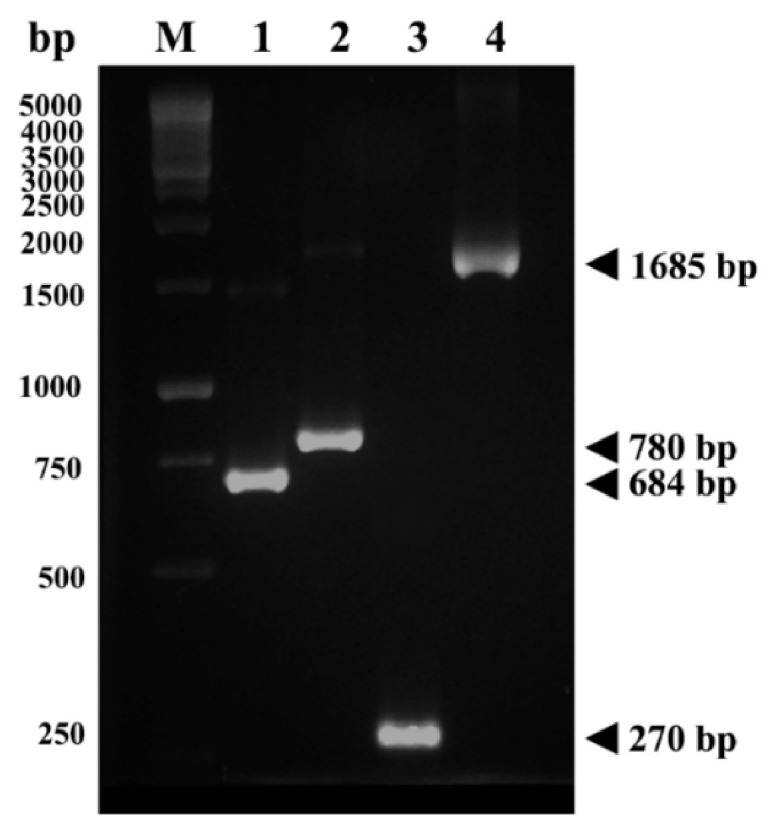
Construction and expression of the rFgCHI-DP chimeric protein. Agarose gel electrophoresis of PCR-amplified gene fragments used for construction of the chimeric gene. Lane M, 1 kb DNA ladder; Lane 1, FgCL1 (684 bp); Lane 2, FgCB1 (780 bp); Lane 3, FgSAP2 (270 bp); Lane 4, assembled chimeric gene FgCHI-AD (1685 bp).

The adult-stage chimeric gene, designated FgCHI-AD, was successfully constructed by combining three antigen genes from *F. gigantica:* cathepsin L1 (FgCL1), cathepsin B1 (FgCB1), and saposin-like protein 2 (FgSAP2). The individual fragments of FgCL1, FgCB1, and FgSAP2 were successfully amplified at the expected sizes of 684, 780, and 270 bp, respectively (Figure 2). The three fragments were subsequently fused to generate the full-length FgCHI-AD construct in the order FgCL1–FgCB1–FgSAP2. The assembled product showed a clear band of approximately 1685 bp on agarose gel electrophoresis, confirming the successful construction of the chimeric gene (Figure 1).

The amplified FgCHI-AD fragment was subsequently cloned into the pGEM-T Easy vector and transformed into *Escherichia coli* DH5α. Recombinant colonies were screened by colony PCR, and plasmids containing the insert were further confirmed by DNA sequencing. Sequence analysis verified the successful assembly of the chimeric construct and confirmed the correct in-frame fusion of the three antigen genes.

### 2.3. Expression and Purification of the rFgCHI-DP Protein

**Figure 3:**
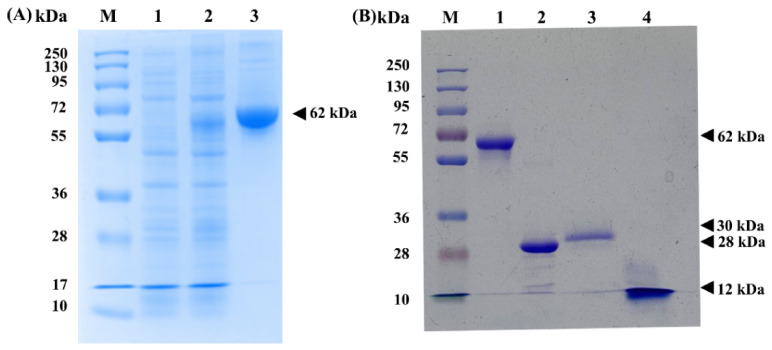
Expression of recombinant rFgCHI-DP and its single protein components. (**A**) SDS-PAGE analysis of rFgCHI-DP expression in *Escherichia coli* BL21. Lane M, protein molecular weight marker; Lane 1, non-induced bacterial lysate; Lane 2, bacterial lysate after induction with 1 mM IPTG for 2 h; Lane 3, purified recombinant rFgCHI-DP showing a protein band at approximately 62 kDa. (**B**) SDS-PAGE analysis of purified recombinant proteins. Lane M, protein molecular weight marker; Lane 1, rFgCHI-DP approximately 62 kDa; Lane 2, rFgCL1 approximately 28 kDa; Lane 3, rFgCB1 approximately 30 kDa; Lane 4, rFgSAP2 approximately 12 kDa.

The recombinant chimeric gene FgCHI-DP (1685 bp) was successfully subcloned into the pET-30b expression vector and transformed into *E. coli* BL21 cells for protein expression. Following induction with IPTG, the recombinant protein was expressed in bacterial cells. SDS-PAGE analysis of the induced bacterial lysate revealed a prominent protein band corresponding to the expected molecular weight of approximately 62 kDa (Figure 3A), which is consistent with the predicted size of the rFgCHI-DP chimeric protein. The expressed recombinant protein was predominantly detected in the insoluble fraction, indicating that rFgCHI-DP was mainly produced in the form of inclusion bodies. The recombinant protein was subsequently purified using Ni-NTA affinity chromatography under denaturing conditions with 8 M urea. After purification, a distinct protein band corresponding to approximately 62 kDa was observed on SDS-PAGE, confirming successful expression and purification of the recombinant chimeric protein rFgCHI-DP. The purified recombinant proteins were analyzed by SDS-PAGE (Figure 3B). The recombinant chimeric protein (rFgCHI-DP) migrated as a single band with an apparent molecular weight of approximately 62 kDa. The individual recombinant proteins, rFgCL1, rFgCB1, and rFgSAP2, were detected at approximately 28, 30, and 12 kDa, respectively.

### 2.4. Antibody Response Evaluation by ELISA

**Figure 4:**
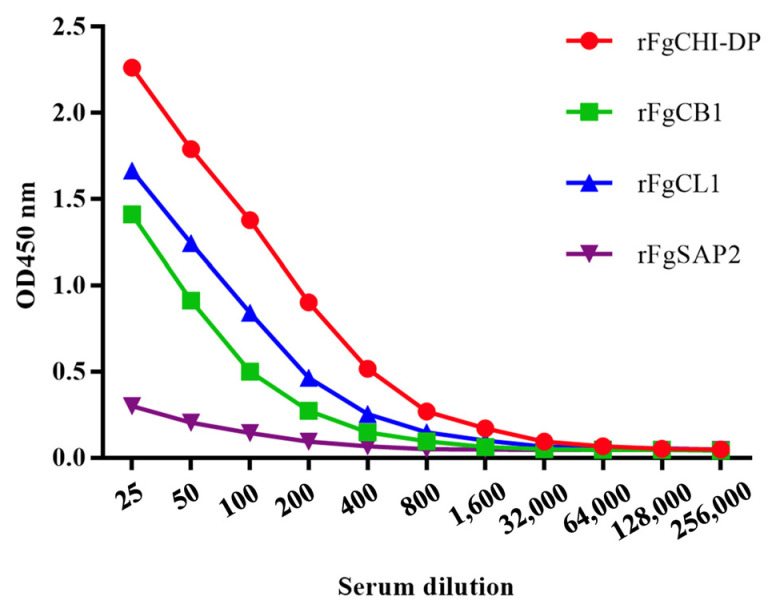
Antigen-specific recombinant protein serum IgG responses. End-point titrations of anti-rFgCHI-DP IgG (red), anti-rFgCB1 IgG (green), anti-rFgCL1 IgG (blue), and anti-rFgSAP2 IgG (purple) antibodies in pooled terminal sera from rFgCHI-DP-immunized mice.

Total IgG levels were measured in rFgCHI-DP-immunized mice with Quil A adjuvant using indirect ELISA in triplicate. The OD_450_ values of rFgCHI-DP-specific IgG in pre-immunized sera were at background levels. The OD_450_ values of IgG against rFgCL1, rFgCB1, and rFgCHI-DP were higher than those observed in the control group. In contrast, the OD_450_ values of IgG against rFgSAP2 showed only a slight increase compared with pre-immunized sera (Figure 4).

### 2.5. Detection of Native Proteins by Western Blot Analysis

**Figure 5:**
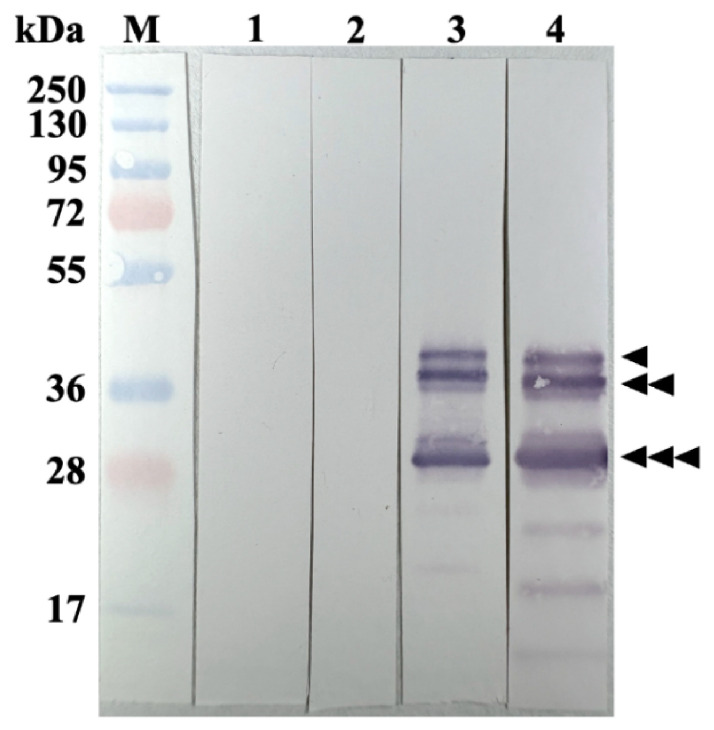
Western blot analysis of native *F. gigantica* antigens recognized by anti-rFgCHI-DP antibodies. Lane M, protein molecular weight marker; Lane 1, metacercariae; Lane 2, newly excysted juveniles (NEJs); Lane 3, 4-week juveniles; Lane 4, adult worms. Anti-rFgCHI-DP antibodies reacted mainly with proteins from the 4-week juvenile and adult stages, producing bands at approximately 36 and 28 kDa. No clear reactivity was observed in metacercariae or NEJ extracts. Arrowheads indicate the reactive bands.

**Figure 6:**
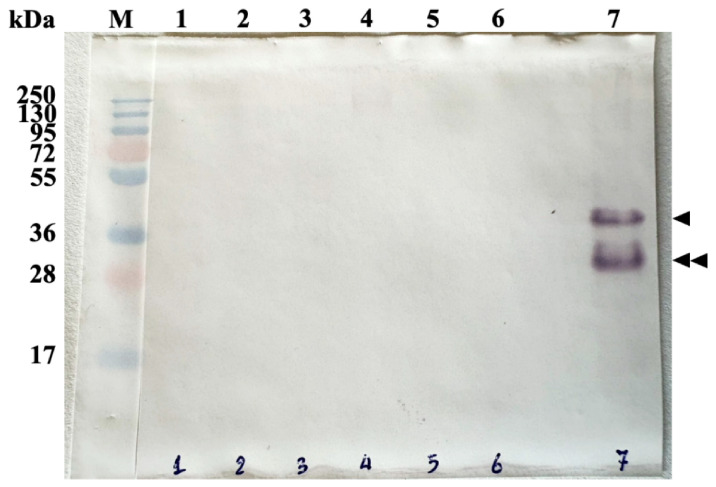
Cross-reactivity analysis of anti-rFgCHI-DP antibodies with proteins from other helminth parasites. Protein extracts from different parasite species were analyzed by Western blot using mouse sera raised against rFgCHI-DP. Lane M, protein molecular weight marker; Lane 1, *P. cervi*; Lane 2, *G. explanatum*; Lane 3, *C. cotylophorum*; Lane 4, *S. labiato-papillosa*; Lane 5, *G. crumenifer*; Lane 6, *E. pancreaticum*; Lane 7, *F. gigantica*. Immunoreactive bands were detected only in the *F. gigantica* extract at approximately 36 kDa and 28 kDa (arrowheads), while no detectable signals were observed in the other parasite extracts, indicating limited cross-reactivity of anti-rFgCHI-DP antibodies.

The ability of antibodies raised against rFgCHI-DP to recognize native parasite antigens was evaluated by Western blot analysis using protein extracted from *F. gigantica* at different developmental stages. Distinct immune-reactive bands were detected in the juvenile and adult parasite extracts, whereas no detectable bands were observed in the metacercariae or newly excysted juvenile stages. The reactive bands were observed at approximately 36 kDa and 28 kDa, corresponding to the expected molecular sizes of native cathepsin proteases (Figure 5). The strongest signals were detected in extracts from the 4-week juvenile and adult stages, suggesting that these antigens are predominantly expressed during later stages of parasite development. To further evaluate the specificity of the antibody response, cross-reactivity of anti-rFgCHI-DP sera with protein extracts from other trematode parasites was examined. Among the tested parasites, immunoreactive bands were detected only in the extract of *F. gigantica*, whereas no detectable signals were observed in extracts from *Paramphistomum cervi*, *Gigantocotyle explanatum*, *Cotylophoron cotylophorum*, *Setaria labiato*-*papillosa*, *Gastrothylax crumenifer* or *Eurytrema pancreaticum*. The observed bands in *F*. *gigantica* were located at approximately 36 kDa and 28 kDa, consistent with the molecular weights of native antigens corresponding to the chimeric protein components (Figure 6).

These results demonstrate that antibodies generated against rFgCHI-DP are capable of recognizing native *F*. *gigantica* proteins, particularly those expressed in the later developmental stages of the parasite, while showing limited cross-reactivity with other trematode species.

### 2.6. Immunolocalization of F. gigantica Tissue

**Figure 7:**
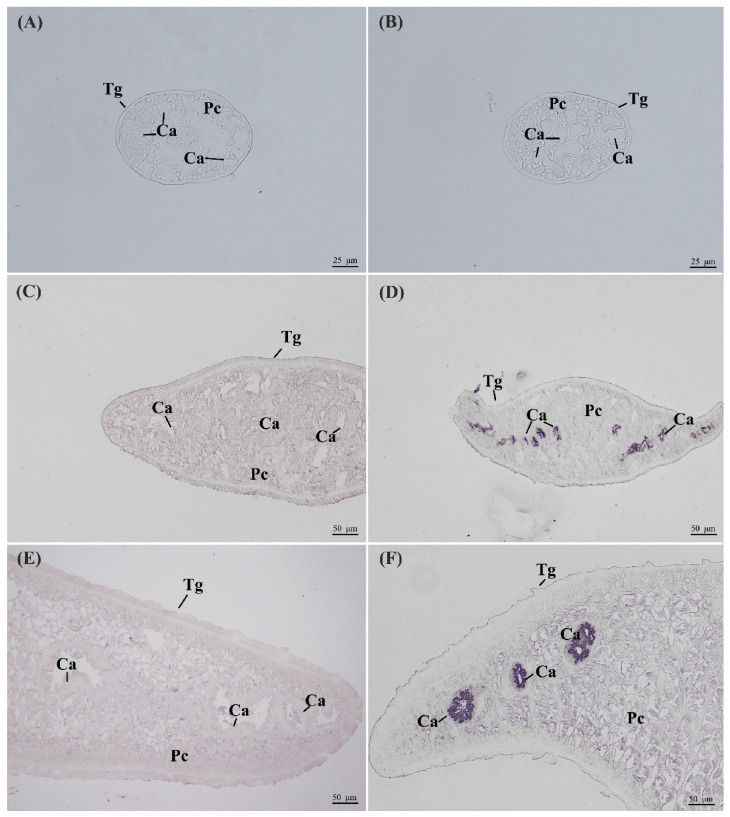
Immunolocalization of native *F. gigantica* antigens recognized by anti-rFgCHI-DP antibodies. (**A**,**B**) Tissue sections of newly excysted juveniles (NEJs); (**C**,**D**) 4-week juveniles; (**E**,**F**) adult worms. Sections incubated with pre-immune mouse serum served as negative controls (**A**,**C**,**E**). Specific immunostaining was observed in the caecal epithelial cells (Ca) of the 4-week juvenile and adult stages (**D**,**F**), whereas no detectable staining was observed in NEJ sections. Tg, tegument; Pc, parenchymal cells; Ca, caecal epithelium.

Immunolocalization was performed to examine the distribution of native *F*. *gigantica* antigens recognized by anti-rFgCHI-DP antibodies. Tissue sections from NEJ, 4-week juveniles, and adult worms were incubated with sera from rFgCHI-DP-immunized mice. No specific staining was observed in NEJ sections, whereas clear immunostaining was detected in the caecal epithelial cells of 4-week juveniles and adult worms. The staining was mainly localized to the epithelial lining of the intestinal caeca, with little or no signal in the tegument or surrounding parenchymal tissues. Sections incubated with pre-immune serum showed no detectable staining (Figure 7). These findings indicate that the antigens recognized by anti-rFgCHI-DP antibodies are primarily associated with the digestive tissues of *F. gigantica*, particularly the caecal epithelium.

## 3. Discussion

Fasciolosis is still an important parasitic disease in livestock, especially in tropical and subtropical areas where *F*. *gigantica* is common [1,2]. Triclabendazole remains the main drug used for treatment, but increasing reports of resistance have raised concerns about relying only on anthelmintic drugs for disease control [3,4,5]. In this study, we constructed an adult-stage chimeric recombinant protein, rFgCHI-DP, composed of FgCL1, FgCB1, and FgSAP2, and evaluated its ability to induce antibody responses in BALB/c mice. The selection of FgCL1, FgCB1, and FgSAP2 was based on their known biological relevance in *Fasciola* infection. Cathepsin L and cathepsin B proteases are major molecules found in the parasite digestive tract and are involved in several important processes, including tissue penetration, feeding, hemoglobin digestion, and immune modulation [5,6,7,8,9,10]. Saposin-like proteins have also been reported to participate in membrane lysis and tissue degradation, which may support parasite survival within the host [7,11,12,13]. This strategy has also been explored in other parasitic diseases, including schistosomiasis, leishmaniasis, and opisthorchiasis [23,24,25], where multi-antigen chimeric proteins have been used to broaden immune recognition and improve vaccine practicality. For example, a chimeric tetraspanin–leucine aminopeptidase subunit vaccine against *Opisthorchis viverrini* provided partial protection in experimentally infected hamsters [26]. Therefore, combining these three proteins into one chimeric construct was expected to provide broader antigenic recognition than a single recombinant protein alone.

The rFgCHI-DP protein was successfully expressed in *E*. *coli* BL21 and purified under denaturing conditions. Most of the protein was found in the insoluble fraction, suggesting that it was mainly produced as inclusion bodies. This result was not surprising, as large recombinant fusion proteins often show poor solubility when expressed in bacterial systems [20,21]. The fusion of several antigenic regions into one molecule may affect protein folding and increase aggregation. Even so, the purified protein was still able to induce antibodies that recognized native parasite proteins. This suggests that important antigenic regions, most likely linear epitopes, were still preserved after purification.

Immunization with rFgCHI-DP formulated with Quil A induced clear antigen-specific IgG responses in mice. Antibody responses against rFgCL1 and rFgCB1 were stronger than those against rFgSAP2. A similar pattern was observed in our previous juvenile-stage chimeric protein study, in which the SAP component also elicited weaker antibody responses than the cathepsin components [22]. The differential antibody responses observed among the three antigenic components may be partially explained by differences in their predicted B-cell epitope content. In silico analysis identified 9 linear B-cell epitopes in CL1 and 12 epitopes in CB1, whereas only a single linear B-cell epitope was predicted in SAP2. This substantial disparity in epitope density is consistent with the stronger IgG responses elicited against CL1 and CB1 and the comparatively weak reactivity observed against SAP2. A greater number of accessible B-cell epitopes may increase the likelihood of B-cell recognition and antibody production, thereby contributing to the immunodominance of CL1 and CB1 within the chimeric construct.

Nevertheless, epitope number alone may not fully explain the observed differences. Previous studies have demonstrated that immune responses to chimeric antigens can be influenced by epitope arrangement, antigen positioning, and linker design, all of which may affect protein folding, domain stability, and epitope accessibility [26,27,28]. Consequently, the relatively weak response to SAP2 may also reflect reduced exposure of SAP2-derived epitopes, structural constraints within the fusion protein, differences in antigen processing and presentation, or lower intrinsic immunogenicity of the native protein. Because balanced immune recognition of all antigenic components is desirable in multivalent vaccine design, further studies incorporating structural analyses, epitope mapping, and alternative antigen arrangements will be valuable for determining whether the SAP2 component can be presented more effectively. Although SAP2 elicited lower antibody responses, its inclusion may still be advantageous by broadening the antigenic repertoire targeted by vaccine-induced immunity.

Western blot analysis showed that anti-rFgCHI-DP antibodies recognized native antigens mainly in the 4-week juvenile and adult stages of *F*. *gigantica*. In contrast, little or no reactivity was detected in metacercariae or newly excysted juveniles. The bands detected at approximately 36 and 28 kDa are within the expected size range of native cathepsin proteins. Since these bands were mainly observed in 4-week juvenile and adult extracts, the antibody response induced by rFgCHI-DP appears to recognize antigens associated with later parasite development rather than the early invasive stages. These findings are consistent with the design rationale of rFgCHI-DP, which was developed to incorporate antigens expressed across the juvenile-to-adult developmental stages of *F. gigantica*. Unlike juvenile-focused vaccine candidates, rFgCHI-DP appears to induce antibodies that preferentially recognize antigens expressed after the parasite has developed beyond the earliest invasive stage. Adult flukes are not only the final stage of infection but also the stage responsible for maintaining chronic disease, continued feeding, egg production, and environmental contamination [1,2,29]. Therefore, immune targeting of adult-associated molecules may not necessarily prevent early invasion, but it could still affect parasite survival, feeding activity, or reproductive output [7,19,29]. Even partial reductions in adult worm survival or egg production may be useful, as they could help reduce disease burden and transmission [29,30].

The cross-reactivity results also showed a good level of specificity. Anti-rFgCHI-DP antibodies reacted with *F*. *gigantica* extracts but showed no clear reaction with the other trematode parasites tested in this study. This is an encouraging finding because vaccine candidate antigens should ideally induce responses that are directed mainly against the target parasite. Although some helminth proteins, especially proteases, may share conserved regions, the antibody response induced by rFgCHI-DP appeared to recognize antigenic determinants that were more specific to *F*. *gigantica*. The immunolocalization results further supported the Western blot findings. Antigens recognized by anti-rFgCHI-DP antibodies were mainly detected in the caecal epithelium of 4-week juvenile and adult flukes, while no staining was observed in the tegument or parenchymal tissues. This localization is consistent with the role of cathepsin proteases as digestive-associated molecules in *Fasciola* species [8,9]. The strong staining in the caecal epithelium suggests that these antigens are linked to feeding and nutrient acquisition, which are important for parasite growth and survival. Antibody responses against these proteins may therefore have the potential to interfere with digestive processes and could potentially affect parasite survival within the host.

Many vaccine studies against fasciolosis have focused on juvenile-stage antigens because early migrating parasites are closely related to tissue invasion and acute liver damage [17,18,22]. However, adult flukes are also important because they are responsible for chronic infection, continued feeding, egg production, and long-term liver pathology. In endemic areas, animals may become infected before completing a vaccination program. For this reason, antigens expressed during later developmental stages may be useful as additional vaccine targets. Juvenile-to-adult stage-associated antigens, such as FgCL1, FgCB1, and FgSAP2, may contribute to broader immune recognition across multiple developmental stages of the parasite and could complement vaccine strategies that primarily target early-stage infection. The use of chimeric proteins is an interesting approach for fasciolosis vaccine development because several antigenic components can be combined into one recombinant molecule [20,21]. This strategy may simplify vaccine production and allow the immune system to recognize more than one parasite target at the same time.

In the present study, rFgCHI-DP was able to induce antibodies that recognized native parasite antigens, supporting the value of this chimeric design. However, immunogenicity alone is not enough to confirm vaccine efficacy. Challenge studies are still needed to determine whether the antibody responses induced by rFgCHI-DP can reduce worm burden, liver pathology, or egg production. Compared with our previous juvenile-stage chimeric protein, which was designed to target antigens associated with early parasite development, rFgCHI-DP showed a recognition pattern more restricted to later developmental stages. This difference supports the idea that chimeric constructs can be designed to direct immune recognition toward specific phases of parasite development. However, there are some limitations to this study. First, the work focused only on immunogenicity and did not include parasite challenge. Second, only antibody responses were evaluated, while cellular immune responses were not examined. Third, the protein was purified under denaturing conditions, which may have affected some conformational epitopes. Future work should therefore test the protective efficacy of rFgCHI-DP in an infection model, evaluate cellular immune responses, and consider alternative expression systems that may improve protein folding and solubility.

Overall, this study shows that rFgCHI-DP is immunogenic and can induce antibodies that recognize native *F. gigantica* antigens expressed mainly in the later stages of parasite development. These findings indicate that juvenile-to-adult stage-associated chimeric proteins can induce antigen-specific immune responses and may serve as a basis for future studies investigating protective efficacy and the development of multistage vaccine strategies against fasciolosis.

## 4. Materials and Methods

### 4.1. B-Cell Epitope Prediction

Linear B-cell epitopes of the 560-amino-acid chimeric protein were predicted using the BepiPred-2.0 server provided by the IEDB Analysis Resource (http://tools.iedb.org/bcell/, accessed on 17 June 2026). The prediction was performed using the default parameters, and residues with scores greater than 0.5 were considered potential B-cell epitopes.

### 4.2. Construction of Recombinant F. gigantica Chimeric Genes FgCatL1, FgCatB1, and FgSAP2

The genes encoding cathepsin L1 (FgCL1), cathepsin B1 (FgCB1), and saposin-like protein 2 (FgSAP2) of *F*. *gigantica* were used as templates for the construction of the adult-stage chimeric gene. The mature regions of FgCL1 and FgCB1 were amplified, while the FgSAP2 sequence excluding the signal peptide region was amplified using gene-specific primers. The amplified fragments were designed with overlapping sequences to allow fusion of the three genes through overlap extension PCR. PCR amplification was performed using Platinum^TM^ Pfx DNA polymerase (Invitrogen, Waltham, MA, USA) under the following conditions: an initial denaturation at 94 °C for 5 min, followed by 35 cycles of denaturation at 94 °C for 15 s, annealing at 55 °C for 30 s, and extension at 68 °C for 2 min, with a final extension at 68 °C for 5 min. The PCR products were separated on a 1% agarose gel and purified using a GeneJET Gel Extraction Kit (Thermo Scientific, Waltham, MA, USA).

**Figure 8:**
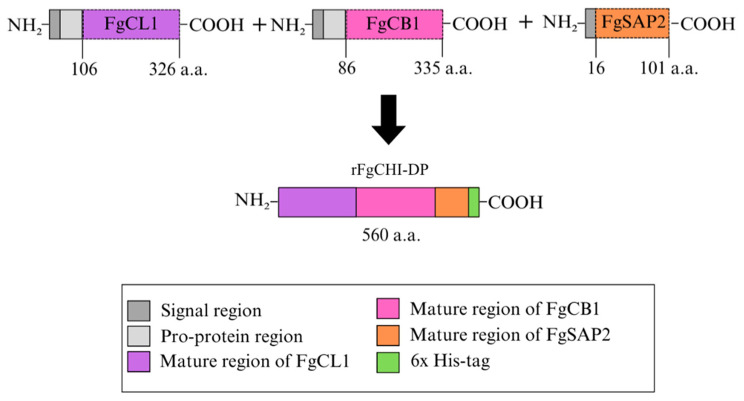
Schematic representation of the rFgCHI-DP with the mature amino sequence of FgCL1, FgCB1 and the full amino sequence of FgSAP2 used in the construction of this protein.

For the fusion step, the purified PCR products were used as templates in an overlap extension PCR to generate the chimeric gene (FgCHI-AD), in which the sequences of FgCL1, FgCB1, and FgSAP2 were sequentially linked. The resulting chimeric PCR product was ligated into the pGEM-T Easy cloning vector (Promega, Fitchburg, WI, USA) and transformed into competent *E*. *coli* DH5α cells. Recombinant colonies were screened by colony PCR, and plasmids containing the correct insert were purified and confirmed by DNA sequencing (Macrogen, Seoul, Rebulic of Korea). Sequence alignment was performed using ClustalW (version 2.1) to verify the integrity of the chimeric gene (Figure 8).

### 4.3. Expression and Purification of Recombinant Proteins (rFgCHI-DP, rFgCL1, rFgCB1, rFgSAP2)

The confirmed FgCHI-AD gene was digested with the restriction enzymes NdeI and NotI and subsequently subcloned into the pET-30b expression vector (Novagen, Madison, WI, USA), which allows expression of the recombinant protein with an N-terminal His-tag. The recombinant plasmid was transformed into competent *E*. *coli* BL21 cells for protein expression. A single colony of transformed bacteria was cultured in LB broth containing kanamycin (100 µg/mL) at 37 °C with shaking at 250 rpm. When the optical density at 600 nm reached approximately 0.6, protein expression was induced by adding isopropyl β-D-1-thiogalactopyranoside (IPTG) to a final concentration of 1 mM. The culture was further incubated for 2 h at 37 °C.

Bacterial cells were harvested by centrifugation at 4000× *g* for 30 min and resuspended in lysis buffer. The recombinant protein was purified using Ni-NTA affinity chromatography under denaturing conditions with 8 M urea. The purified protein was concentrated using Amicon Ultra centrifugal filters with a 10 kDa molecular weight cutoff (Millipore, Burlington, MA, USA). The purified recombinant protein, designated rFgCHI-DP, had an approximate molecular weight of 62 kDa and was stored at −80 °C until further use.

### 4.4. Immunization Protocol

**Figure 9:**
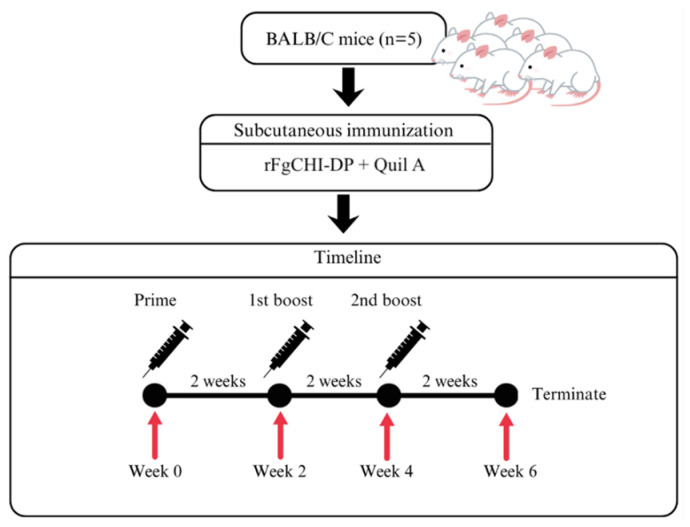
Experimental design of mouse immunization with rFgCHI-DP.

Five-week-old male BALB/c mice (*n* = 5) were maintained under standard laboratory conditions with ad libitum access to food and water. All experimental procedures were conducted in accordance with institutional guidelines for animal care and were approved by the Animal Ethics Committee of Burapha University. The rFgCHI-DP-immunized mice were subcutaneously injected with recombinant rFgCHI-DP formulated with Quil A adjuvant. The primary immunization was administered at week 0, followed by booster immunizations at weeks 2 and 4. Each mouse received 50 µg of recombinant protein mixed with 15 µg of Quil A adjuvant for the primary immunization, and 25 µg of recombinant protein with the same amount of adjuvant for each booster dose. Pre-immunized sera were used as negative controls. Blood samples were collected from all animals at two-week intervals (weeks 0, 2, 4, and 6). Serum samples were separated by centrifugation and stored at −20 °C until use (Figure 9).

### 4.5. ELISA Analysis

All individual serum samples were analyzed for IgG levels using an indirect ELISA in triplicate. Briefly, 96-well microtiter plates were coated with 100 µL of 0.05 M carbonate–bicarbonate buffer (pH 9.6) containing 1 µg/mL of recombinant proteins (rFgCHI-DP, rFgCB1, rFgCL1, and rFgSAP2) and incubated overnight at 4 °C. Following incubation, the plates were washed three times with phosphate-buffered saline (PBS; 140 mM NaCl, 2.7 mM KCl, 10 mM Na_2_HPO_4_, 1.8 mM KH_2_PO_4_, pH 7.4) containing 0.05% Tween-20 (PBST). The plates were then blocked with 1% bovine serum albumin (BSA) for 2 h at room temperature (RT) to prevent nonspecific binding, followed by three washes with PBST. Serum samples collected from immunized mice were diluted 1:1000 in PBS. Subsequently, 50 µL of two-fold serially diluted serum was added to each well and incubated at 37 °C for 1 h. After washing the plates three times with PBST, 50 µL of goat anti-mouse IgG conjugated with horseradish peroxidase (HRP), diluted 1:5000 in PBS, was added and incubated at 37 °C for 1 h. The plates were then washed three times with PBST, followed by the addition of 50 µL of 3,3′,5,5′-tetramethylbenzidine (TMB) substrate (KPL, Gaithersburg, MD, USA) to each well. The reaction was incubated for 15 min at RT in the dark and subsequently stopped by adding 50 µL of 1 N HCl. Finally, the optical density (OD) was measured at 450 nm using an automatic spectrophotometer (Flow Laboratories, McLean, VA, USA).

### 4.6. Western Blotting Analysis

Western blot analysis was performed to determine whether antibodies raised against the rFgCHI-DP could recognize native antigens of *F*. *gigantica* at different developmental stages and to evaluate potential cross-reactivity with other trematode parasites. Whole-body extracts of *F*. *gigantica* at different developmental stages, including metacercariae, newly excysted juveniles (NEJs), 4-week juveniles, and adult worms, were prepared by homogenization in lysis buffer containing Tris–HCl, NaCl, Triton X-100, and EDTA. The homogenates were sonicated and subsequently centrifuged at 12,000× *g* for 1 h at 4 °C. The supernatants containing soluble parasite proteins were collected and stored at −80 °C until use. Protein samples (5 µg per lane) were separated by 12.5% SDS-PAGE and transferred onto nitrocellulose membranes using a semi-dry blotting system. After transfer, membranes were blocked with 5% skim milk in phosphate-buffered saline containing 0.1% PBST for 1 h at RT to reduce nonspecific binding.

The membranes were incubated with mouse sera obtained from rFgCHI-DP-immunized mice diluted 1:500 in PBST containing 1% skim milk for 1 h at RT. Pre-immune mouse serum was used as a negative control. Following incubation, membranes were washed three times with PBST and then incubated with alkaline phosphatase-conjugated goat anti-mouse IgG (Invitrogen, Waltham, MA, USA) diluted 1:2000 for 1 h. Protein bands were visualized using nitro blue tetrazolium/5-bromo-4-chloro-3-indolyl phosphate (NBT/BCIP) substrate solution. The enzymatic reaction was stopped using stop buffer (PBS containing 20 mM EDTA), and membranes were subsequently scanned and documented.

To investigate antibody cross-reactivity, whole-body extracts of other trematode parasites [31], including *P. cervi*, *G. explanatum*, *C. cotylophorum*, *S. labiato-papillosa*, *E. pancreaticum*, and *F. gigantica*, were subjected to SDS-PAGE and Western blotting under the same conditions described above.

### 4.7. Immunohistochemistry Analysis

Immunolocalization analysis was conducted to determine the distribution of target antigens recognized by anti-rFgCHI-DP antibodies in *F*. *gigantica* tissues. Parasites at different developmental stages, including newly excysted juveniles (NEJs), four-week-old juveniles (4WKJ), and adult flukes, were fixed and embedded in paraffin. Tissue sections of approximately 5 µm thickness were prepared and mounted on glass slides.

The sections were deparaffinized and rehydrated through graded ethanol series. Antigen retrieval was performed by heating the sections in citrate buffer (pH 6.0) using a microwave oven. After cooling, nonspecific binding sites were blocked with 4% BSA in PBS for 1 h at RT. The sections were then incubated with mouse serum obtained from rFgCHI-DP-immunized mice (pooled serum) diluted 1:500 in PBS for 1 h at RT. Pre-immune serum was used as a negative control. After washing with PBS, the sections were incubated with alkaline phosphatase-conjugated goat anti-mouse IgG (Invitrogen, USA) diluted 1:1000 for 1 h. Color development was performed using NBT/BCIP substrate solution. The reaction was stopped with stop buffer, and the sections were examined under a light microscope. Images were captured to evaluate the localization of parasite antigens recognized by the anti-rFgCHI-DP antibodies.

## 5. Conclusions

This study showed that rFgCHI-DP, composed of FgCL1, FgCB1, and FgSAP2, could be successfully produced and used to induce specific antibody responses in mice. The antibodies generated against rFgCHI-DP recognized native *F. gigantica* antigens, mainly in the 4-week juvenile and adult stages, and localized to the caecal epithelium of the parasite. These results suggest that rFgCHI-DP contains antigenic regions relevant to parasite development from the juvenile to adult stages. Although challenge experiments are still required to confirm protective efficacy, this construct may serve as a foundation for further investigation of multistage vaccine strategies against fasciolosis.

## Figures and Tables

**Table 1:** Predicted B-cell epitopes on a sequence of rFgCHI-DP.

No.	Start	End	Peptide	Protein
1	7	11	KIDWR	FgCL1
2	15	25	YVTEVKDQGNC	FgCL1
3	34	38	TGTME	FgCL1
4	41	44	YMKN	FgCL1
5	61	67	GPWGNMG	FgCL1
6	86	96	ETESSYPYTAV	FgCL1
7	130	136	GAEGPAA	FgCL1
8	173	178	GTQGGT	FgCL1
9	187	193	WGSSWGE	FgCL1
10	226	236	SFDAREKWPNC	FgCB1
11	239	248	ISEIPDQSSC	FgCB1
12	269	275	NGEKKPR	FgCB1
13	291	299	GCEGGYPSM	FgCB1
14	313	323	GTLENPTGCLP	FgCB1
15	331	341	HLEETPGLAPC	FgCB1
16	344	346	ELY	FgCB1
17	350	354	KFEKQ	FgCB1
18	356	379	QAGYSKTHEEDKIKGKSSYNVGDR	FgCB1
19	441	448	SWNEGWGE	FgCB1
20	460	462	ECG	FgCB1
21	473	482	SFDVPSKQKN	FgCB1
22	545	546	QH	FgSAP2

## Data Availability

The original contributions presented in this study are included in the article. Further inquiries can be directed to the corresponding author.

## References

[1] Mas-Coma S., Valero M.A., Bargues M.D. (2019). Fascioliasis. Adv. Exp. Med. Biol..

[2] Meemon K., Sobhon P. (2015). Juvenile-specific cathepsin proteases in *Fasciola* spp.: Their characteristics and vaccine efficacies. Parasitol. Res..

[3] Sanabria R., Ceballos L., Moreno L., Romero J., Lanusse C., Alvarez L. (2013). Identification of a field isolate of *Fasciola hepatica* resistant to albendazole and susceptible to triclabendazole. Vet. Parasitol..

[4] Kelley J.M., Elliott T.P., Beddoe T., Anderson G., Skuce P., Spithill T.W. (2016). Current threat of triclabendazole resistance. Trends. Parasitol..

[5] Dalton J.P., Robinson M.W., Mulcahy G., O’Neill S.M., Donnelly S. (2013). Immunomodulatory molecules of *Fasciola hepatica*: Candidates for both vaccine and immunotherapeutic development. Vet. Parasitol..

[6] Meemon K., Grams R., Vichasri-Grams S., Hofmann A., Korge G., Viyanant V., Upatham E.S., Habe S., Sobhon P. (2004). Molecular cloning and analysis of stage and tissue-specific expression of cathepsin B encoding genes from *Fasciola gigantica*. Mol. Biochem. Parasitol..

[7] Kueakhai P., Changklungmoa N., Chaichanasak P., Jaikua W., Itagaki T., Sobhon P. (2015). Vaccine potential of recombinant pro- and mature cathepsinL1 against fasciolosis gigantica in mice. Acta. Trop..

[8] Robinson M.W., Dalton J.P., Donnelly S. (2008). Helminth pathogen cathepsin proteases: It’s a family affair. Trends Biochem. Sci..

[9] Cwiklinski K., Dalton J.P., Dufresne P.J., Course J.L., Williams D.J., Hodgkinson J., Paterson S. (2015). The *Fasciola hepatica* genome: Gene duplication and polymorphism reveals adaptation to the host environment and the capacity for rapid evolution. Genome Biol..

[10] Dalton J.P., Brindley P.J., Knox D.P., Brady C.P., Hotez P.J., Donnelly S., O’Neill S.M., Mulcahy G., Loukas A. (2003). Helminth vaccines: From mining genomic information for vaccine targets to systems used for protein expression. Int. J. Parasitol..

[11] Grams R., Adisakwattana P., Ritthisunthorn N., Eursitthichai V., Vichasri-Grams S., Viyanant V. (2006). The saposin-like proteins 1, 2, and 3 of *Fasciola gigantica*. Mol. Biochem. Parasitol..

[12] Caban-Hernandez K., Espino A.M. (2013). Differential expression and localization of saposin-like protein 2 of *Fasciola hepatica*. Acta Trop..

[13] Espino A.M., Hillyer G.V. (2003). Molecular cloning of a member of the *Fasciola hepatica* saposin-like protein family. J. Parasitol..

[14] Kueakhai P., Changklungmoa N., Waseewiwat P., Thanasinpaiboon T., Cheukamud W., Chaichanasak P., Sobhon P. (2017). Characterization and vaccine potential of *Fasciola gigantica* saposin-like protein 1 (SAP-1). Vet. Parasitol..

[15] Kueakhai P., Changklungmoa N., Chaithirayanon K., Phatsara M., Preyavichyapugdee N., Riengrojpitak S., Sangpairoj K., Chusongsang P., Sobhon P. (2015). Saposin-like protein 2 has an immunodiagnostic potential for detecting fasciolosis gigantica. Exp. Parasitol..

[16] Mirzadeh A., Valadkhani Z., Yoosefy A., Babaie J., Golkar M., Esmaeili Rastaghi A.R., Kazemi-Rad E., Ashrafi K. (2017). Expression, purification and in vitro refolding of the recombinant truncated saposin-like protein 2 antigen for development of diagnosis of human fascioliasis. Acta. Trop..

[17] Wesołowska A., Basałaj K., Norbury L.J., Sielicka A., Wędrychowicz H., Zawistowska-Deniziak A. (2018). Vaccination against *Fasciola hepatica* using cathepsin L3 and B3 proteases delivered alone or in combination. Vet. Parasitol..

[18] Villa-Mancera A., Alcalá-Canto Y., Olivares-Pérez J., Molina-Mendoza P., Hernández-Guzmán K., Utrera-Quintana F., Carreón-Luna L., lmedo-Juárez A., Reynoso-Palomar A. (2021). Vaccination with cathepsin L mimotopes of *Fasciola hepatica* in goats reduces worm burden, morphometric measurements, and reproductive structures. Microb. Pathog..

[19] Kueakhai P., Changklungmoa N., Cheukamud W., Osotprasit S., Chantree P., Preyavichyapugdee N., Sobhon P., Meemon K. (2021). The combined recombinant cathepsin L1H and cathepsin B3 vaccine against *Fasciola gigantica* infection. Parasitol. Int..

[20] de Oliveira N.R., Jorge S., Gomes C.K., Rizzi C., Pacce V.D., Collares T.F., Monte L.G., Dellagostin O.A. (2017). A novel chimeric protein composed of recombinant *Mycoplasma hyopneumoniae* antigens as a vaccine candidate evaluated in mice. Vet. Microbiol..

[21] Strohl W.R. (2015). Fusion proteins for half-life extension of biologics as a strategy to make biobetters. BioDrugs.

[22] Cheukamud W., Chansap S., Rattanasroi K., Changklungmoa N., Kueakhai P. (2024). Construction and mouse antibody response evaluation of juvenile stage-specific chimeric protein from *Fasciola gigantica*. Vet. Parasitol..

[23] Pinheiro C.S., Ribeiro A.P.D., Cardoso F.C., Martins V.P., Figueiredo B.C.P., Assis N.R.G., Morais S.B., Caliari M.V., Loukas A., Oliveira S.C. (2014). A multivalent chimeric vaccine composed of *Schistosoma mansoni* SmTSP-2 and Sm29 was able to induce protection against infection in mice. Parasite Immunol..

[24] Lage D.P., Ribeiro P.A.F., Dias D.S., Mendonça D.V.C., Ramos F.F., Carvalho L.M., Oliveira D., Steiner B.T., Martins V.T., Perin L. (2020). A candidate vaccine for human visceral leishmaniasis based on a specific T cell epitope-containing chimeric protein protects mice against *Leishmania infantum* infection. npj Vaccines.

[25] Phung L.T., Chaiyadet S., Hongsrichan N., Sotillo J., Hang D.T.D., Canh T.Q., Brindley P.J., Loukas A., Laha T. (2020). Partial protection with a chimeric tetraspanin-leucine aminopeptidase subunit vaccine against *Opisthorchis viverrini* infection in hamsters. Acta. Trop..

[26] Orbegozo-Medina R.A., Martínez-Sernández V., Folgueira I., Mezo M., González-Warleta M., Perteguer-Prieto M.J., Romarís F., Leiro J.M., Ubeira F.M. (2019). Antibody responses to chimeric peptides derived from parasite antigens in mice and other animal species. Mol. Immunol..

[27] Chen X., Zaro J.L., Shen W.C. (2013). Fusion protein linkers: Property, design and functionality. Adv. Drug Deliv. Rev..

[28] Reddy Chichili V.P., Kumar V., Sivaraman J. (2013). Linkers in the structural biology of protein–protein interactions. Protein. Sci..

[29] Lalor R., Cwiklinski K., Calvani N.E.D., Dorey A., Hamon S., Corrales J.L., Dalton J.P., De Marco Verissimo C., De Waal T., Skuce P.J. (2021). Pathogenicity and virulence of the liver flukes *Fasciola hepatica* and *Fasciola gigantica* that cause the zoonosis fasciolosis. Virulence.

[30] Turner J., Howell A., McCann C., Caminade C., Bowers R.G., Williams D., Baylis M. (2016). A model to assess the efficacy of vaccines for control of liver fluke infection. Sci. Rep..

[31] Changklungmoa N., Kueakhai P., Apisawetakan S., Riengrojpitak S., Sobhon P., Chaithirayanon K. (2014). Identification and expression of *Fasciola gigantica* thioredoxin. Parasitol. Res..

